# The burden of stroke and transient ischemic attack in Pakistan: a community-based prevalence study

**DOI:** 10.1186/1471-2377-9-58

**Published:** 2009-12-01

**Authors:** Ayeesha Kamran Kamal, Ahmed Itrat, Muhammed Murtaza, Maria Khan, Asif Rasheed, Amin Ali, Amna Akber, Zainab Akber, Naved Iqbal, Sana Shoukat, Farzin Majeed, Danish Saleheen

**Affiliations:** 1 Stroke Service, Section of Neurology, Department of Medicine, Aga Khan University Hospital, Karachi, Pakistan; 2 Medical College, Aga Khan University Hospital, Karachi, Pakistan; 3 Department of Biological and Biomedical Sciences, Aga Khan University Hospital, Karachi, Pakistan; 4 Dow University of Health Sciences, Karachi, Pakistan; 5 Department of Public Health and Primary Care, University of Cambridge, UK

## Abstract

**Background:**

The burden of cerebrovascular disease in developing countries is rising sharply. The prevalence of established risk factors of stroke is exceptionally high in Pakistan. However, there is limited data on the burden of stroke and transient ischemic attack (TIA) in South Asia. We report the first such study conducted in an urban slum of Karachi, Pakistan.

**Methods:**

Individuals 35 years of age or older were invited for participation in this investigation through simple random sampling. A structured face-to-face interview was conducted using a pre-tested stroke symptom questionnaire in each participant to screen for past stroke or TIA followed by neurological examination of suspected cases. Anthropometric measurements and random blood glucose levels were recorded. Multivariable logistic regression was used to determine the association of vascular risk factors with prevalence of stroke.

**Results:**

Five hundred and forty five individuals (49.4% females) participated in the study with a response rate of 90.8%. One hundred and four individuals (19.1%) were observed to have a prior stroke while TIA was found in 53 individuals (9.7%). Overall, 119 individuals (21.8% with 66.4% females) had stroke and/or TIA. Female gender, old age, raised random blood glucose level and use of chewable tobacco were significantly associated with the prevalence of cerebrovascular disease.

**Conclusion:**

This is the first study demonstrating an alarmingly high life-time prevalence of cerebrovascular disease in Pakistan. Individual and public health interventions in Pakistan to increase awareness about stroke, its prevention and therapy are warranted.

## Background

The risk of stroke has increased by 100% in low and middle income countries over the last decade and the developing world accounts for 85.5% of mortality due to all stroke deaths worldwide[1] Socioeconomic transition in low and middle income countries is likely to increase the burden of cerebrovascular disease[2] Patients who suffer from stroke in countries such as Pakistan are almost a decade younger than their western counterparts and thus, the disability in stroke survivors and resulting economic losses may be greater[3]

The prevalence of modifiable risk factors for stroke in the Pakistani population is alarmingly high. Hypertension affects one in three adults aged greater than 45 years and 19% of the population aged 15 years and above[4] The National Health Survey of Pakistan showed that diabetes mellitus is present in 35% of people older than 45 years[4] The overall prevalence of obesity is 28% in women and 22% in men while the prevalence of tobacco use is 33% in men and 4.7% in women[5,6] Given the wide prevalence of risk factors, the burden of stroke in Pakistan is likely to be substantial.

Transient Ischemic Attack (TIA) defines a subset of patients prone to stroke who may benefit from timely intervention. The immediate risk of stroke is about 10% in the first 90 days after a TIA with 50% of this risk in the first 48 hours[7] Data from Pakistan also supports these observations[8] Early intervention after TIA has shown an 80% relative risk reduction in the emergence of stroke in western cohorts[9,10] This clinically recognizable syndrome is an important pragmatic entry point for stroke prevention in those at highest risk of "conversion"[11]

No multiethnic prevalence studies of stroke or TIA have been reported from Pakistan to date. The present study reports the life-time prevalence of stroke and TIA and their associated modifiable risk factors in a multiethnic urban population.

## Methods

### Study Population

A randomized community-based cross-sectional survey was conducted between September 2008 and January 2009 in Bilal Colony which is an urban slum in Karachi, the largest metropolitan city of Pakistan. This community has a history of interaction with the Aga Khan University (AKU) through a community outreach center established by our collaborators in Department of Pediatrics and Child Health[12]

According to a recent census (August 2008) conducted by the same department, the population of Bilal Colony is 76,361 individuals in 10,925 households with an average monthly household income equivalent to $124 ± $93 US dollars. In context, Gross National Income (GNI) per capita per annum in Pakistan is $980 US dollars[13] Urdu, Punjabi, Baluchi and Pushto are the major languages spoken in this multiethnic population and more than 99% people are Muslims. Roughly 50% have received no education while 10% have received religious education only.

### Study Design

Census data was used to generate a simple random list of households where individuals above the age of 35 years resided. Community health workers (CHWs, trained volunteers with at least 6 years of work experience in Bilal Colony) visited the selected households to invite eligible individuals for face-to-face interviews. Single individuals from each household who met the inclusion criteria were invited. An additional batch of multilingual community volunteers accompanied the team to provide ease of conversation. Invitations along with an information leaflet were provided at these home visits.

All men and women aged 35 years or above who were residents of Bilal Colony for two years or more were eligible for the study. Upon non-availability or refusal by an individual, the next household on the list was approached. Recruitment was continued for a total of 15 days until the targeted sample size was reached.

### Sample Size Estimation

Using a 95% confidence, 5% estimated stroke prevalence reported previously and 2% bounds of error; a required sample size of 457 participants was calculated. Six hundred households were approached during the study period. In order to establish if our sample was representative of the community at large, age and gender distribution of our participants was compared with that of the census data.

### Data Collection and Diagnosis

**Figure 1:**
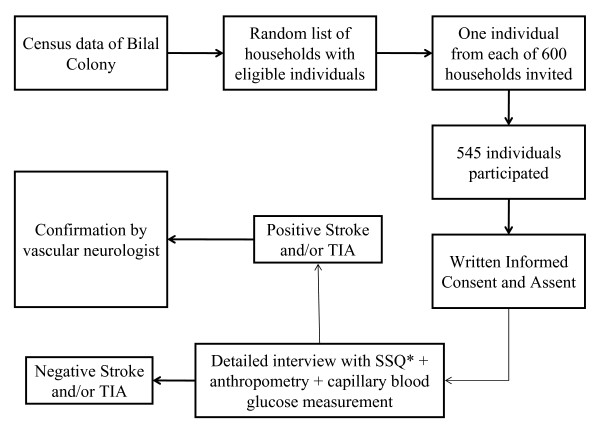
**Overview of the study design**. *SSQ = Stroke Symptom Questionnaire.

A study clinic was organized by this project team at the Maternal and Child Health Clinic, Bilal Colony. This clinic was divided into three areas - Interview (Area I), Neurological Assessment (Area II) and Physical Examination, Anthropometry and Measurements (Area III). Logistics were designed to facilitate automatic transfer from Area I through III of each participant. Each participant would register at Area I. After explanation of the study procedure and written informed consent, all participants underwent a structured interview with physicians trained in using a standardized questionnaire. This questionnaire was based on the Stroke Symptom Questionnaire (SSQ) and the TIA Symptom Questionnaire, which was translated into Urdu by three independent translators and a final version was selected after group review[14] The questionnaire was divided into the following sections: 1) socio-demographics, 2) stroke symptoms ever experienced, 3) TIA symptoms experienced in the last 12 months, 4) known risk factors. Pre-testing of the questionnaire was carried out on 35 individuals with a similar age and socioeconomic status distribution as the study population. A copy of the questionnaire can be found here (English: Additional file 1; Urdu: Additional file 2). All participants were shown photographs depicting amaurosis fugax, diplopia, triplopia (triple vision; perception of three images of a single object), hemianopia, hemiparesis and facial paresis as a part of their interview (see Additional file 3). Following the interview, any cases suspected to have had stroke or TIA were examined and confirmed by a vascular neurologist on site (Area II). Stroke and TIA were defined using published criteria[15,16] Anthropometric measurements and random blood glucose levels using Abbott MediSense Optium Glucose Monitor were recorded in all participants. All instruments used for anthropometric measurements were calibrated on a daily basis. The study protocol was approved by the Ethical Review Committee at Aga Khan University. Informed consent and verbal assent was given by all participants prior to the interview. Participants who met the eligibility criteria but were unable to travel to the study site due to financial or physical reasons were provided transport. Interviews were also conducted on holiday weekends to ensure the participation of daily wage workers without any financial repercussions. No financial incentives were provided to any study participant. An overview of the study design is presented in figure 1.

### Statistical Analysis

All data was entered twice by two individuals separately and was cross-checked to ensure that no errors were made during the process. Prevalence estimates of stroke, and transient ischemic attack were reported with 95% confidence intervals (CI). Vascular risk factors between various groups were compared using univariate logistic regression to calculate odds ratios and 95% CIs. Variables significant at p < 0.1 were included in the final multivariable logistic regression model. Data was analyzed using Stata version 10.0 (StataCorp. LP, TX, USA)

## Results

A total of 545 individuals including 49.4% females took part in the study with a response rate of 90.8%. As compared to the community census data of individuals older than 35 years, we had a slightly higher proportion of females in our sample (49.4% in this sample v/s 43.9% in the census, p = 0.012). The mean age ± standard deviation (SD) of responders was 48.7 ± 12.8 years while that found in the census data was 46.6 ± 11.1 years (p < 0.001). Characteristics of the study population have been described in table 1.

**Table 1:** Socio-demographic characteristics of the study population (n = 545)

	Males(n = 276)	Females(n = 269)	Total (n = 545)
	**N (%)**	**n(%)**	**n(%)**

**Age (years)**			

35 - 45	123(44.6)	164(61.0)	287(52.7)

46 - 55	52(18.8)	64(23.8)	116(21.3)

56 - 65	48(17.4)	29(10.8)	77(14.1)

65 - 75	33(12.0)	10(3.7)	43(7.9)

More than 75	20(7.2)	2(0.7)	22(4.0)

**Ethnicity**			

Pathan	91(33.0)	58(21.6)	149(27.3)

Punjabi	72(26.1)	71(26.4)	143(26.2)

Sindhi	52(18.8)	37(13.8)	89(16.3)

Balochi	3(1.1)	6(2.2)	9(1.7)

Afghan	3(1.1)	19(17.1)	22(4.0)

Others	55(19.9)	79(29.0)	133(24.4)

**Education**			

None	136(49.3)	148(55.0)	284(52.1)

Religious only	36(13.0)	82(30.5)	118(21.7)

Some school	103(37.3)	39(14.5)	142(26.1)

**Marital Status**			

Married	261(95.6)	221(82.5)	482(88.4)

Single	7(2.6)	8(3.0)	15(2.8)

Divorced/Widowed	5(1.9)	39(14.6)	44(8.1)

**Figure 2:**
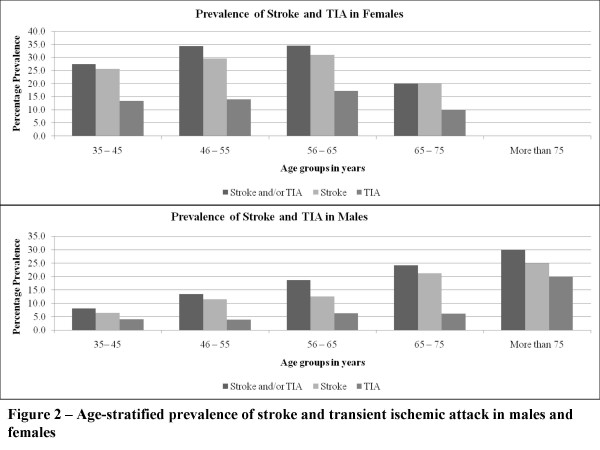
**Age-stratified prevalence of cerebrovascular disease in males and females: the life-time prevalence of CVD in females was similar in all age groups (p = 0.611) while that in males rose with old age (p = 0.025)**. *TIA = Transient Ischemic Attack.

Stroke was reported in 104 individuals (19.1% with 69.2% female) while TIA was reported in 53 individuals (9.7% with 69.8% female). Overall, stroke and/or TIA were found in 119 individuals (21.8% with 66.4% females). The prevalence of stroke and TIA across gender, age groups and ethnicities has been shown in table 2. Age-stratified prevalence of stroke and TIA in males and females is presented in figure 2.

**Table 2:** Life-time prevalence of stroke and transient ischemic attack (TIA) in population sub-groups

	Stroke (n = 104)		TIA (n = 53)		Stroke/TIA (n = 119)	
	**n**	**% (95% CI)**	**n**	**% (95% CI)**	**n**	**% (95% CI)**

**Gender**						

Male	32	11.6 (7.8-15.4)	16	5.8 (3.0-8.6)	40	14.5 (10.3-18.7)

Female	72	26.8 (21.4-32.1)	37	13.7(9.6-17.9)	79	29.4 (23.9-34.8)

**Age (years)**						

35 - 45	50	17.4 (13.2-22.3)	27	9.4 (6.3-13.4)	55	19.2 (14.8-24.2)

46 - 55	25	21.6 (14.5-30.1)	11	9.5 (4.1-14.9)	29	25.0 (17.4-33.9)

56 - 65	15	19.5 (11.3-30.1)	8	10.4 (3.4-17.4)	19	24.7 (15.6-35.8)

66 - 75	9	20.9 (10.0-36.0)	3	7.0 (1.5-19.1)	10	23.3 (11.8-38.6)

More than 75	5	22.7 (7.8-45.4)	4	18.2 (5.2-40.3)	6	27.2 (10.7-50.2)

**Ethnicity**						

Pathan	24	16.1 (10.6-23.0)	13	8.7 (4.7-14.5)	29	19.5 (13.4-26.7)

Punjabi	27	18.9 (12.8-26.3)	17	11.9 (7.1-18.4)	32	22.4 (15.8-30.1)

Sindhi	16	18.0 (10.6-27.5)	10	11.2 (5.5-19.7)	18	20.2 (12.4-30.1)

Balochi	4	44.4 (13.7-78.8)	2	22.2 (2.8-60.0)	4	44.4 (13.7-78.8)

Afghan	9	41.0 (20.7-63.6)	5	22.7 (7.8-45.4)	9	31.0 (20.7-63.6)

Others	24	18.0 (11.9-25.6)	6	4.5 (1.7-9.6)	27	20.3 (13.8-28.1)

						

**Total**	104	19.1 (15.9-22.6)	53	9.7 (7.4-12.5)	119	21.8 (18.4-25.5)

Note: TIA - transient ischemic attack, CI - confidence interval

Female gender (p < 0.001), family history of coronary artery disease or stroke (p = 0.013), elevated random blood sugar (p = 0.004), hypertension (p = 0.002), body mass index ≥25 kg/m^2 ^(p = 0.006) and past history of myocardial infarction (p = 0.023) were significantly associated with risk of stroke and/or TIA on univariate analysis. In the multivariable model, old age, female gender, family history of coronary artery disease or stroke, elevated random blood sugar and use of chewable tobacco products were significantly associated with risk of stroke and/or TIA. Univariate and multivariable analysis of risk factors is presented in table 3 and table 4 respectively.

**Table 3:** Risk factor profile and non-adjusted Odds Ratios for stroke and/or transient ischemic attack.

	No CVD	Stroke/TIA	Non-adjusted odds ratio	P-value
	**n (%)^a^**	**n (%)^a^**	**OR (95% CI)**	

**Age, years (SD)**	48.3 (12.7)	50.0 (12.8)	1.01 (0.99-1.03)	0.192

**Female gender**	190 (44.6)	79 (66.4)	2.45 (1.60-3.75)	< 0.001

**Family history of CAD or Stroke**	86 (20.2)	37 (31.1)	1.78 (1.13-2.81)	0.013

**Past Myocardial Infarction**	24 (5.6)	11 (11.8)	2.23 (1.12-4.47)	0.023

**Systolic BP, mmHg (SD)**	128.6 (21.0)	134.9 (27.3)	1.06 (1.02-1.11)^b^	0.008

**Diastolic BP, mmHg (SD)**	81.8 (12.1)	84.2 (14.2)	1.08 (0.997-1.167)^b^	0.061

**Hypertension^c^**	203 (47.7)	76 (63.9)	1.94 (1.28 - 2.95)	0.002

**Random Blood Glucose, mg/dL (SD)**	141.6 (69.6)	164.1 (86.9)	1.02 (1.005-1.030)^2^	0.004

**Elevated RBS^d^**	74 (17.4)	36 (30.3)	2.06 (1.30-3.28)	0.002

**Smoking**	123 (28.9)	25 (21.0)	0.66 (0.40-1.07)	0.090

**Pan, Gutka or Supari Use^e^**	78 (18.3)	30 (25.2)	1.50 (0.93-2.43)	0.096

**Weight, kg (SD)**	67.7 (17.3)	69.0 (19.2)	1.00 (0.99-1.02)	0.468

**Body mass index, kg/m^2 ^(SD)**	26.6 (8.0)	27.5 (6.1)	1.01 (0.99-1.04)	0.269

**Obesity^f^**	229 (53.8)	81 (68.1)	1.83 (1.19-2.82)	0.006

**Waist Circumference, cm (SD)**	93.2 (13.6)	94.7 (16.0)	1.01 (0.99-1.02)	0.301

**Waist-Hip Ratio (SD)**	0.94 (0.09)	0.93 (0.08)	0.46 (0.05-4.72)	0.516

**Elevated WHR^g^**	375 (88.0)	109 (91.6)	1.48 (0.73-3.02)	0.278

**Menopause^h^**	98 (57.7)	47 (62.7)	1.23 (0.71-2.15)	0.462

Note: WHR - Waist-hip Ratio, BMI - Body mass index, RBS - Random Blood Sugar, CAD - Coronary Artery Disease, BP - Blood Pressure, SD - Standard Deviation, TIA - Transient Ischemic Attack, CI - Confidence Interval.

^a^Given as n(%) except where stated specifically.

^b^Calculated for each 5 unit increase.

^c^Defined as systolic BP greater than or equal to 140 mmHg or diastolic BP greater than or equal to 90 mmHg on two readings at least 10 minutes apart and/or self-reported history of persistent hypertension.

^d^Raised Random Blood Sugar level greater than or equal to 180 mg/dL on one reading.

^e^Pan, gutka and supari are locally available forms of chewable tobacco.

^f^Defined as body mass index more than or equal to 25 kg/m^2^.

^g^Defined as waist-hip ratio greater than 0.88 in males and 0.81 in females.

^h^Menopause was considered in the female subset of our sample only.

**Table 4:** Results of multivariable analysis of significant risk factors for stroke and/or transient ischemic attack.

	No CVD	Stroke/TIA	Adjusted odds ratio	P-value
	**n (%)^a^**	**n (%)^a^**	**OR (95% CI)**	

**Age, years (SD)**	48.3 (12.7)	50.0 (12.8)	1.022 (1.003-1.041)	0.021

**Female gender**	190 (44.6)	79 (66.4)	2.62 (1.56-4.40)	< 0.001

**Family history of CAD or Stroke**	86 (20.2)	37 (31.1)	1.65 (1.02-2.69)	0.042

**Past Myocardial Infarction**	24 (5.6)	11 (11.8)	1.62 (0.77-3.39)	0.205

**Hypertension^b^**	203 (47.7)	76 (63.9)	1.25 (0.78-2.00)	0.504

**Elevated RBS^c^**	74 (17.4)	36 (30.3)	1.76 (1.07-2.90)	0.026

**Smoking**	123 (28.9)	25 (21.0)	1.03 (0.58-1.83)	0.921

**Pan, Gutka or Supari Use**	78 (18.3)	30 (25.2)	2.06 (1.22-3.49)	0.007

**Obesity^d^**	229 (53.8)	81 (68.1)	1.49 (0.92-2.41)	0.104

Note: RBS - Random Blood Sugar, CAD - Coronary Artery Disease, BP - Blood Pressure, SD - Standard Deviation, TIA - Transient Ischemic Attack, CI - Confidence Interval. Variables biologically plausible or significant at p < 0.100 on univariate analysis were included in this model.

^a^Given as n(%) except where stated specifically.

^b^Defined as systolic BP greater than or equal to 140 mmHg or diastolic BP greater than or equal to 90 mmHg on two readings at least 10 minutes apart and/or self-reported history of persistent hypertension.

^c^Raised Random Blood Sugar level greater than or equal to 180 mg/dL on one reading.

^d^Defined as body mass index more than or equal to 25 kg/m^2^.

Of the patients who reported stroke or TIA, 7% reported residual disability (Modified Rankin Score2-4) whereas 93% were symptom-free (Modified Rankin Score 0).

## Discussion

This study is the first urban population based estimate of life-time prevalence of cerebrovascular disease in Pakistan that shows an alarmingly high burden of disease. In comparison to existing worldwide literature on stroke prevalence, this study shows a prevalence of stroke which is almost twice the highest reported prevalence in the world to date (table 5)[17,18] A prior study reported a stroke prevalence of 4.8% in a single ethnic group in Pakistan[4] However, the definition of stroke used in this study was limited to the presence of sustained hemiparesis for more than 24 hours. Given the wide variety of presentations of stroke, it is likely to be an underestimate of the true burden. In comparison, we report about 1 in 5 people in our sample have suffered from stroke or TIA. In addition, our data also suggests that patients who have suffered from stroke in Pakistan have an average age of about 50 years which is almost a decade younger than their western counterparts. A previous study from Pakistan has reported about 25% prevalence of coronary artery disease in a middle-aged sample[19] Hence, our results reiterate the exceptionally high burden of atherosclerotic diseases in Pakistan. In addition, family history of coronary artery disease increased the risk of stroke and/or TIA by at least 32% in our sample. These findings highlight the shared genetic and environmental etiologies that may have a role in atherosclerotic vascular disease in the South Asian population.

**Table 5:** Comparison of worldwide prevalence of stroke over the last 20 years

Author	Method of Diagnosis of Stroke	Study Method	Sample Population	Year	Important Findings
Bharucha et al[24]	Clinical diagnosis by a neurologist	Population-based door-to-door survey	India, Bombay (n = 14 010)	1988	Crude prevalence was 842 per 100 000 population; age-specific rates were higher in men

Mittelmark et al[25]	Self-reported history plus medical record confirmation	Population based longitudinal study	Four regions, USA (n = 5,201)	1989-90	Crude prevalence rate was 246 per 100,000a

Bots et al[26]	Self-reported history plus medical record confirmation	Population-based, cohort	Rotterdam, Netherlands (n = 7983)	1990-93	A total of 352 individuals out of 7983 were reported to have a stroke, while an additional 285 were reported with clinical data. This represents a crude prevalence rate of 7979 per 100,000a

Geddes et al[27]	Self-reported stroke questionnaire through postal service	Population based, point prevalence study	Yorkshire, UK (n = 18,827)	1991	Crude prevalence rate was 4680 per 100,000, with males having a higher prevalence

Bonita et al[28]	Clinical diagnosis using WHO definition	Retrospective analysis of hospital, clinical and autopsy record	Auckland, New Zealand (n = 854000 and 945 000)^b^	1991-92	Age-adjusted rate was 833 per 100, 000

Prencipe et al[29]	Self-reported history followed by neurological examination	Community-based, door-to-door survey	L'Aquila, Italy (n = 1032)	1992	Crude prevalence rate was 7300 per 100,000. Prevalence of stroke was higher in men and increased with age in both sexes

O'Mahony et al[30]	Screening questionnaire followed by clinical confirmation using WHO criteria	Population based, point prevalence study	Newcastle, UK (n = 2000)	1993	Crude prevalence rate was 4740 per 100,000, while age adjusted rates were 1750 per 100,000. Prevalence increased proportionately in older age groups

Huang et al[31]	unclear	Population-based, Cross sectional??	Taiwan, China (n = 11, 925)	1994	Crude prevalence rate was 595 per 100,000

Nicoletti et al[32]	WHO Stroke screening instrument	Population based door-to-door survey	Cordillera, Bolivia (n = 9955)	1994	Crude prevalence rate was 663 per 100,000 for those >/= 35 years. Prevalence in men was 2× greater than women

Banergee et al[33]	Clinical diagnosis by a neurologist or CT imaging	Population-based cluster survey	India, Calcutta (n = 50 291)	1998-1999	Crude prevalence was 147 and age-adjusted rate was 334 per 100 000 population; females had higher prevalence in all age groups

Anand et al[34]	Self-reported history or clinical diagnosis by physician	Population-based cross-sectional	Canada (n = 985)	2000	Crude prevalence rates were similar among ethnic groups: South Asians: 300, European whites: 1800, and Chinese: 600 per 100 000 population

AASAP[35]	Unclear	Based on national health records of individual country	Nine Asian countries (Pakistan was not part of this study)	2000	Crude prevalence in India ranges from 90-222 per 100 000; Thailand and Taiwan had higher reported prevalence rates (690 and 1430) per 100 000

Jafar et al[4]	Self-reported history	Community survey and target sampling	Pakistan (n = 500)	2001	Crude prevalence was 4800 per 100 000

Venketa-subramanium et al[36]	Clinical diagnosis using WHO definition	Population-based, cross-sectional	Singapore (n = 15 606)	2001-2003	Crude as well as age-standardized rates were similar among ethnic groups (SA: 362, Malays: 332, Chinese: 376) per 100 000 population

Department of Health Survey for England[37]	Clinical diagnosis using WHO definition	Population-based door-to-door health survey	Stratified proportionate sample from general population	2005	Crude prevalence in South Asians (Indian: 1100, Pakistani: 1800, Bangladeshi: 1800) were lower than European Whites (2400) per 100 000 population

This Study - Kamal et al	Self-reported history based on SSQ followed by neurological examination	Community-based following census	Karachi, Pakistan (n = 545)	2008-2009	Crude prevalence was determined to be 19000 per 100,000. Women found to have a higher prevalence of stroke and at an earlier age than men.

WHO: World Health Organization

SA: South Asian

SSQ: Stroke Symptom Questionnaire

^a ^Calculated using information from the publication cited

^b^two separate studies done ten years apart

We further suggest that females are at a higher risk of stroke and/or transient ischemic attack in our population and this finding is independent of all other risk factors. Females were well-represented in this sample as the study site was visited by women frequently and they did not require male chaperones to participate in the study. Hence, the design of this study ensured appropriate participation of females, a usually under-represented group.

The age standardized prevalence of stroke and TIA followed a predictable rise with old age in men while it was evenly distributed across all age groups in females (figure 2). These findings suggest gender specific susceptibility to etiologies in addition to atherosclerosis. Further studies looking at specific vascular risk factors unique to females are needed. These studies may be focused on eclampsia, cerebral venous sinus thrombosis, gestational diabetes and stroke - these are beyond the scope of the current study[20] Since 70% of all births are at home and attended by traditional birth attendants, such studies require a community based design[21]

Uncontrolled diabetes and use of chewable tobacco were two modifiable risk factors independently associated with stroke and/or TIA. About 1 in 3 stroke patients were found to have uncontrolled diabetes which conferred at least a 7% increased risk of stroke and/or TIA after adjustment for all other risk factors. Overall, the prevalence of raised random blood sugar was 20% which is lower than but comparable to the prevalence of diabetes reported earlier[4] Similarly, 1 in 4 patients used chewable tobacco in the form of pan, *gutka *or *supari *(locally available forms with areca and betel nut) which increased their risk of stroke and/or TIA by at least 22% after adjustment. The overall prevalence of chewable tobacco was similar to that reported by an earlier survey conducted in the same community[22]

Hypertension was found in about 50% of all participants while increase in each 5 mmHg of systolic blood pressure significantly increased the risk of stroke and/or TIA by at least 2% on univariate analysis. Hypertension, as defined by elevated systolic or diastolic blood pressure or self-reported past medical history of hypertension, was significantly associated with stroke and/or TIA on univariate analysis but did not reach statistical significance in the final adjusted model. About 57% of the participants were obese with body mass index ≥25 kg/m^2 ^and obesity increased the risk of stroke and/or TIA by at least 19% on univariate analysis.

We confirm the association of known modifiable risk factors with cerebrovascular disease in Pakistan. Similar reports of high burden of cardiovascular risk factors have been published earlier [4-6] Our data also shows that only 19% of stroke patients in this study were aware of their condition while about 58% of all participants with elevated blood pressures were aware that they had hypertension. These findings highlight the need for community education about modifiable vascular risk factors. They also call attention to the role that general practitioners can play in our setup through promotion of lifestyle modifications and through aggressive therapeutic control of diabetes and hypertension in order to lower the risk of stroke and TIA in the community.

The strengths of this study include standardization using a pre-tested questionnaire, on site verification of diagnosis by a vascular neurologist, a random sample from a multiethnic population and appropriate arrangements for representation of women and daily wage workers. The use of a combination of questions to assess stroke and TIA prevalence has been shown to improve sensitivity at the risk of false positives[23] We also used photographs of the experience of visual symptoms of stroke and TIA to assist in clear diagnosis. For example, participants could clearly identify historically whether they had "non-specific clouding of vision" vs. "hemianopia" by looking at a photograph depicting hemianopia. While this approach may have increased the specificity of the visual section of our questionnaire, we have not measured this effect.

Our sample size may have limited power to study independent associations with all possible risk factors. We were unable to confirm an association with smoking perhaps because the population prefers indigenous means of tobacco intake over cigarette smoking. As compared to the census data, mean age of our sample around two years greater and we had a slightly higher representation of females. However, the prevalence of vascular risk factors in our sample is similar to that reported earlier in literature. Hence, we believe that the findings of this study are reliable and a call for action.

Since the site of the study was located in an urban slum of Pakistan with a predominantly low socioeconomic status, the results of this study may not be completely applicable in the rural areas of the country where the prevalence may be lower as "demographic transition" may not have occurred. However, they may be applied in similar socioeconomic strata in Pakistan.

## Conclusion

We report an alarmingly high burden of stroke and TIA in the urban Pakistani population. Our findings mimic the reported prevalence of cardiovascular disorders and their risk factors in Pakistan[4,19] The association of female gender and local chewable tobacco use with stroke begs further investigation. Community awareness about stroke and its modifiable risk factors was limited. Individual and public health interventions in Pakistan to increase awareness of stroke, its prevention and therapy are warranted.

## Source of funding

This study was funded by the University Research Council at Aga Khan University Grant Number: URC Project ID 07GS021MED. This is part of the "small grants program".

## Competing interests

The authors declare that they have no competing interests.

## Authors' contributions

AK conceived the study, supervised data collection and wrote the manuscript. AI performed study coordination, field management and was involved in all stages of the project including writing the grant. MM, MK, AR, AA, AA, ZA, NI, SS, FM performed field work, manuscript writing and data entry. MM and AI performed statistical analysis. DS generated a randomization list and provided statistical overview. All authors have read and reviewed the final manuscript.

## Pre-publication history

The pre-publication history for this paper can be accessed here:

http://www.biomedcentral.com/1471-2377/9/58/prepub

## Supplementary Material

Additional file 1 **Supplement 1**. The Burden of Stroke and Transient Ischemic Attacks in Pakistan: a Community-based Prevalence Study (English). Click here for file

Additional file 2 **Supplement 2**. The Burden of Stroke and Transient Ischemic Attacks in Pakistan: a Community-based Prevalence Study (Urdu). Click here for file

Additional file 3 **Supplement 3**. This PDF contains the visual aids used in the study. Click here for file
