# Supplementary material for: The burden of stroke and transient ischemic attack in Pakistan: a community-based prevalence study
Source: BMC Neurol. 2009 Dec 1;9:58. doi: 10.1186/1471-2377-9-58 (PMC2793240; doi:10.1186/1471-2377-9-58)
Supplement: Additional file 1 — Supplement 1. The Burden of Stroke and Transient Ischemic Attacks in Pakistan: a Community-based Prevalence Study (English). [file 1471-2377-9-58-S1.PDF]

The Burden of Stroke and Transient Ischemic Attacks in Pakistan: a Community-based Prevalence Study

Instructions:

1. *Separate form should be filled in for each subject*
2. *Introduce yourself and explain the purpose of your interview*
3. *Verbal consent should be obtained from the respondent before the interview*

Registration no. ☐☐☐

Questionnaire

Section I: Demographics:

**S.no**	
1.1 Respondent's name	
1.2 House No:	
1.3 Telephone No	
1.4 This questionnaire is being answered by	Respondent:____ Other (Specify):____________
1.5 Date of birth	___dd/_____mm/________yy
1.6 Age:	
1.5 Gender:	Male: ☐ Female: ☐
1.7 Ethnicity	Pathan☐ Punjabi☐ Balochi☐<br>Sindhi☐ Afghan☐ Other (*Specify)*☐<br>_________________
1.8 Handedness:	Right☐ Left☐
1.9 Education:	Illiterate☐<br>Schooling☐<br>Specify years:_____
1.10 Profession:	
1.11 Marital status:	Single☐Married☐Divorced☐Widowed☐

Section II: Stroke symptom questionnaire

S.No	SECTION : II<br>Stroke Symptom Questionnaire:	Code List	Code
2.1a)	Have you ever had a weakness or paralysis of a complete body side or an arm or leg?	Yes……………1<br>No…..................2<br>Don't Know…..0	
2.1b)	If Yes, Please briefly describe symptoms and their duration		
2.1c)	Were these symptoms treated by a physician?	Yes…..1, No….2	
2.2 a)	Have you ever had a hanging corner of the mouth that you could not pull back voluntarily?	Yes……………1<br>No…..................2<br>Don't Know…..0	
2.2 b)	If Yes, Please briefly describe symptoms and their duration		
2.2 c)	Were these symptoms treated by a physician?	Yes…..1, No….2	
2.3 a)	Have you ever had a slurred speech or problems to talk to somebody because your mouth was unable to	Yes……………1<br>No…..................2	

	articulate words or sentences correctly?	Don't Know…..0	
2.3 b)	If Yes, Please briefly describe symptoms and their duration		
2.3 c)	Were these symptoms treated by a physician?	Yes…..1, No….2	
2.4a)	Have you ever had numbness or sensory loss of a complete body side or an arm or leg?	Yes……………1<br>No…................2<br>Don't Know…..0	
2.4 b)	If Yes, Please briefly describe symptoms and their duration		
2.4 c)	Were these symptoms treated by a physician?	Yes…..1, No….2	
2.5a)	Have you ever had one or more of the visual symptom on one or both eyes?	Yes……………1<br>No…................2<br>Don't Know…..0	
2.5 b)	If Yes, Please briefly describe symptoms and their duration		
2.5 c)	Were these symptoms treated by a physician?	Yes…..1, No….2	
2.6 a)	Have you ever been diagnosed with stroke by a physician?	Yes……………1<br>No…................2<br>Don't Know…..0	
2.6 b)	If Yes, Please briefly describe symptoms and their duration		
2.6 c)	Were these symptoms treated by a physician?	Yes…..1, No….2	

Section III: TIA symptom Questionnaire

Section III.A- Numbness or tingling

S.no	Question	Code List	Code
3.A.1:	In the past 12 months have you had any sudden feeling of numbness, tingling or loss of feeling in either arm, hand, leg, foot or face? **IF NO GO TO SECTION IIIB.**	Yes……….1<br>No………..2<br>**IF NO, GO TO SECTION IIIB.**	
3.A.2:	Please briefly describe your symptoms:		
3.A.3	Which arm, hand , leg, foot or face was affected? (you may check more than one answer)	Right Face…..1 Left Face……6<br>Right Arm…...2 Left Arm……7<br>Right Hand…..3 Left Hand…..8<br>Right Leg……4 Left Leg…….9<br>Right Foot…..5 Left Foot…..10	
3.A.4	Did you have pain in the arm or leg along with the feeling of numbness?	Yes………1<br>No……….2	
3.A.5	How many attacks of such numbness or tingling have you had ?	Only One……................1<br>Two…………................2<br>Three to Five…………...3<br>> than Five(specify)……4	
3.A.6:	If only one, when did this attack occur? (mm/yy) ____		
3.A.7:	If more than one attack, when was the first attack? (mm/yy)________________		

3.A.8	When was the last attack ?(mm/yy) __________		
3.A.9	If more than one attack, how often did attacks occur?	Usually about once a day……..1<br>Usually several times a day .....2<br>Usually about once a week …..3<br>Usually several times a week…4<br>Usually several times a month .5<br>Usually several times a year….6	
3.A.10:	How long did attacks usually last?	Usually less than 5 minutes…..1<br>From 5 minutes to one hour…..2<br>From 1 to 6 hours……………..3<br>From 6 to 24 hours……………4<br>More than a day………………5	
3.A.11:	Please check any of the following symptoms that may have occurred at about the same time as the numbness or tingling.	Paralysis………………………1<br>Speech disturbance……………2<br>Blackouts or fainting………….3<br>Severe headaches……………...4<br>Visual loss……………………5<br>Convulsions or seizures………6<br>Dizzy or giddy spells…………7<br>Attacks of nervousness ………8	
3.A.12:	Did you see a doctor for the numbness/tingling?	Yes……..1 No………2	

Section III.B- Paralysis

S.no	Question	Code List	Code
3.B.1	In the past 12 months have you had any sudden attacks of paralysis or loss of use of either arm, hand, leg or foot?**IF NO GO TO SECTION IIIC.**	Yes……….1<br>No………..2<br>**IF NO, GO TO SECTION IIIC.**	
3.B.2	Please briefly describe your symptoms:		
3.B.3:	Which arm, hand , leg, foot or face was affected? (you may check more than one answer)	Right Face…..1 Left Face……6<br>Right Arm…...2 Left Arm……7<br>Right Hand…..3 Left Hand…..8<br>Right Leg……4 Left Leg…….9<br>Right Foot…..5 Left Foot…..10	
3.B.4:	Did you have pain in the arm or leg along with the paralysis?	Yes………1<br>No……….2	
3.B.5	: How many attacks of such paralysis have you had ?	Only One…….................1<br>Two…………................2<br>Three to Five…………...3<br>> than Five(specify)……4	
3.B.6:	If only one, when did this attack occur? (mm/yy)____		
3.B.7:	If more than one attack, when was the first attack? (mm/yy)________________		
3.B.8:	When was the last attack ?(mm/yy) __________		
3.B.9:	If more than one attack, how often did attacks occur?	Usually about once a day……..1<br>Usually several times a day .....2<br>Usually about once a week …..3<br>Usually several times a week…4<br>Usually several times a month .5<br>Usually several times a year….6	

3.B.10:	How long did attacks usually last?	Usually less than 5 minutes…..1<br>From 5 minutes to one hour…..2<br>From 1 to 6 hours……………..3<br>From 6 to 24 hours……………4<br>More than a day………………5	
3.B.11:	Please check any of the following symptoms that may have occurred at about the same time as the paralysis.	Speech disturbance……………2<br>Blackouts or fainting…………3<br>Severe headaches……………...4<br>Visual loss……………………5<br>Convulsions or seizures………6<br>Dizzy or giddy spells…………7<br>Attacks of nervousness ………8<br>Numbness or tingling…………9	
3.B.12	: Did you see a doctor for the paralysis?	Yes……..1 No………2	

Section III.C- Vision

S.no	Question	Code List	Code
3.C.1:	In the past 12 months have you had any sudden loss of eyesight or blurring of vision for a short period of time ?<br>**IF NO GO TO SECTION IIID.**	Yes……….1<br>No………..2<br>**IF NO, GO TO SECTION IIID.**	
3.C.2:	Please briefly describe your symptoms:		
3.C.3	What part of your vision was affected?	Right Eye…..1<br>Left Eye …...2<br>Both eyes…...3<br>Vision to the right side…..4<br>Vision to the left side……5	
3.C.4:	How many attacks of loss of eyesight or blurring of vision have you had?	Only One……................1<br>Two…………................2<br>Three to Five…………...3<br>> than Five(specify)……4	
3.C.5	If only one, when did this attack occur? (mm/yy)____		
3.C.6	If more than one attack, when was the first attack? (mm/yy)________________		
3.C.7:	When was the last attack ?(mm/yy)<br>__________		
3.C.8:	If more than one attack, how often did attacks occur?	Usually about once a day……..1<br>Usually several times a day .....2<br>Usually about once a week …..3<br>Usually several times a week…4<br>Usually several times a month .5<br>Usually several times a year….6	
3.C.9:	How long did attacks usually last?	Usually less than 5 minutes…..1<br>From 5 minutes to one hour…..2<br>From 1 to 6 hours……………..3<br>From 6 to 24 hours……………4<br>More than a day………………5	
3.C.10:	Please check any of the following symptoms that may have occurred at about the same time as the visual disturbance.	Paralysis………………………1<br>Speech disturbance……………2<br>Blackouts or fainting…………3<br>Severe headaches……………...4<br>Convulsions or seizures………6<br>Dizzy or giddy spells…………7	

		Attacks of nervousness ………8<br>Numbness or tingling…………9	
3.C.11	Did you see a doctor for the visual disturbance?	Yes……..1 No………2	

Section III.D- Speech

S.no	Question	Code List	Code
3.D.1:	In the past 12 months have you had any sudden changes in speech, loss of speech or inability to say words for more than 2 minutes?**IF NO GO TO SECTION IIIE.**	Yes……….1<br>No………..2<br>**IF NO, GO TO SECTION IIIE.**	
3.D.2:	Please briefly describe your symptoms:		
3.D.3:	How many attacks of loss of speech have you had?	Only One……..................1<br>Two…………................2<br>Three to Five…………...3<br>> than Five(specify)……4	
3.D.4:	If only one, when did this attack occur? (mm/yy)____		
3.D.5	: If more than one attack, when was the first attack? (mm/yy) _____________		
3.D.6:	When was the last attack ?(mm/yy) __________		
3.D.7	: If more than one attack, how often did attacks occur?	Usually about once a day……..1<br>Usually several times a day .....2<br>Usually about once a week …..3<br>Usually several times a week…4<br>Usually several times a month .5<br>Usually several times a year….6	
3.D.8:	How long did attacks usually last?	Usually less than 5 minutes…..1<br>From 5 minutes to one hour…..2<br>From 1 to 6 hours……………..3<br>From 6 to 24 hours……………4<br>More than a day………………5	
3.D.9:	Please check any of the following symptoms that may have occurred at about the same time as the speech difficulty.	Paralysis………………………1<br>Blackouts or fainting…………3<br>Severe headaches……………..4<br>Visual loss…………………….5<br>Convulsions or seizures………6<br>Dizzy or giddy spells…………7<br>Attacks of nervousness ………8<br>Numbness or tingling…………9	
3.D.10	: Did you see a doctor for the speech difficulty?	Yes……..1 No………2	

Section III.E- Dizziness

S.no	Question	Code List	Code
3.E.1	In the past 12 months have you had any spells of dizziness, difficulty in walking, lightheadedness or loss of balance?**IF NO GO TO SECTION IV.**	Yes……….1<br>No………..2<br>**IF NO, GO TO SECTION IV.**	
3.E.2	Please briefly describe your symptoms:		
3.E.3	Please check any of the following symptoms	Dizziness …………………….1	

	which may have occurred.	Loss of balance…....................2<br>Blackouts or fainting…………3<br>Spinning sensation (vertigo)..4<br>Difficulty walking……………5	
3.E.4	How many attacks do you think you have has in the past 12 months ?	Only One……................1<br>Two…………................2<br>Three to Five…………...3<br>> than Five(specify)……4	
3.E.5	If only one, when did this attack occur? (mm/yy)____		
3.E.6	If more than one attack, when was the first attack? (mm/yy)_______________		
3.E.7	When was the last attack ?(mm/yy) _______		
3.E.8	If more than one attack, how often did attacks occur?	Usually about once a day……..1<br>Usually several times a day .....2<br>Usually about once a week …..3<br>Usually several times a week…4<br>Usually several times a month .5<br>Usually several times a year….6	
3.E.9	How long did attacks usually last?	Usually less than 5 minutes…..1<br>From 5 minutes to one hour…..2<br>From 1 to 6 hours……………..3<br>From 6 to 24 hours……………4<br>More than a day………………5	
3.E.10	Please check any of the following symptoms that may have occurred at about the same time as the dizziness, the difficulty in walking, the lightheadedness or the loss of balance.	Paralysis………………………1<br>Speech disturbance……………2<br>Severe headaches……………...4<br>Visual loss…………………….5<br>Convulsions or seizures………6<br>Attacks of nervousness ………8<br>Numbness or tingling…………9<br>Falling down…………………10<br>Staggering on walking ……….11<br>Nausea………………………..12<br>Vomiting……………………...13	
3.E.11	Did you see a doctor for the symptoms?	Yes……..1 No………2	

Section IV - Risk Factor Assessment:

S.no	Past history	Code list	Code
4.1	Have you ever had a stroke?	Yes…1, No…..2	
4.2	Have you ever had a TIA?	Yes…1, No…..2	
4.3	Have you ever had an irregular heart rhythm?	Yes…1, No…..2	
4.4<br>4.5	Are you diabetic?<br>Are you on any medications for diabetes?	Yes…1, No…..2	
4.6	Are you hypertensive? (>140/90. >130/80 in DM)	Yes…1, No…..2	
4.7	Have you ever had your blood pressure checked?		
4.8	If yes, what were the readings? __________		
4.9	Are you on any blood pressure lowering medications?	Yes…1, No…..2	
4.10	Do you smoke?	Yes…1, No…..2	
4.11	If yes, how many pack years?_________		
4.12	If you have quit smoking, how long ago did you quit? ________		
4.13	Do you have dyslipidemia/raised cholesterol?	Yes…1, No…..2	
4.14	Have you ever had seizures?	Yes…1, No…..2	
4.15	Have you ever had chest pain?	Yes…1, No…..2	
4.16	If yes, was the chest pain at rest?	Yes…1, No…..2	

4.17	Was there chest pain on exertion?	Yes…1, No…..2	
4.18	Have you ever had an MI (heart attack)?	Yes…1, No…..2	
4.19	Have you ever had a coronary bypass surgery?	Yes…1, No…..2	
4.20	Have you ever had Rheumatic fever?	Yes…1, No…..2	
4.21	Have you ever had valvular heart disease?	Yes…1, No…..2	
4.22	Have you ever had a valve replacement surgery?	Yes…1, No…..2	
4.23	Do you have pain in your legs on walking?	Yes…1, No…..2	
4.24	If yes, does the pain immediately disappear on rest?	Yes…1, No…..2	
4.25	Do you suffer from this pain every time you walk?	Yes…1, No…..2	
4.26	Do you drink alcohol?	Yes…1, No…..2	
4.27	When did you last taste alcohol?		
4.28	Do you eat *pan/Ghutka/Supari* ?	Yes…1, No…..2	
4.29	Are you post menopausal?	Yes…1, No…..2	
4.30	**Berlin Scale for apnea**		
	Category one		
4.31	Do you snore?	Yes…1, No…2,<br>Don't know….3	
4.32	If you snore, your snoring is:	Slightly louder than breathing…1<br>As loud as talking…2<br>Louder than talking..3<br>Very loud-can be heard in adjacent rooms…4	
4.33	How often do you snore	Nearly everyday…..1<br>3-4 times a week….2<br>1-2 times a week….3<br>1-2 times a month...4<br>Never or nearly never.5	
4.34	Has your snoring ever bothered people	Yes…1, No…2,<br>Don't know….3	
4.35	Has anyone ever noticed that you quit breathing during your sleep?	Nearly everyday…..1<br>3-4 times a week….2<br>1-2 times a week….3<br>1-2 times a month...4<br>Never or nearly never.5	
4.36	Category 2		
4.37	How often do you feel tired or fatigued after your sleep?	Nearly everyday…..1<br>3-4 times a week….2<br>1-2 times a week….3<br>1-2 times a month...4<br>Never or nearly never.5	
4.38	During your waking time, do you feel tired, fatigued or not up to par?	Nearly everyday…..1<br>3-4 times a week….2<br>1-2 times a week….3<br>1-2 times a month...4<br>Never or nearly never.5	
4.39	have you ever nodded off or fallen asleep while driving a vehicle?	Yes…1, No…2	
4.40	How often does this occur?	Nearly everyday…..1<br>3-4 times a week….2<br>1-2 times a week….3<br>1-2 times a month...4<br>Never or nearly never.5	
4.41	Category 3		
4.42	Do you have high blood pressure ?	Yes…1, No…2,	

		Don't know….3	
	**Family History : Check each that applies and mention the youngest age at onset**	**Code List:**<br>**Yes…1**<br>**No….2**<br>**Mother…3**<br>**Father….4**<br>**Brother...5**<br>**Sister…..6**<br>**Second degree rel…7**	**Code/ Age at onset**
4.43	Stroke		
4.45	TIA		
4.46	Diabetes		
4.47	Hypertension		
4.48	Seizures		
4.49	CAD/Angina		
4.50	*Premature CAD		

* Family history of premature CAD (CAD in male first degree relatives ≤ 55 years, in female first degree relatives ≤ 65 years)
First degree relatives= Parents, siblings, offspring
Second degree relatives= Grandparent, aunt, uncle, nephew, niece, half-sibling, grandchild

Section V- Disability Assessment:

level	Description	
0	No symptoms	
1	No significant disability, despite symptoms; able to perform all usual duties and activities	
2	Slight disability; unable to perform all previous activities but able to look after own affairs without assistance	
3	Moderate disability; requires some help, but able to walk without assistance	
4	Moderately severe disability; unable to walk without assistance and unable to attend to own bodily needs without assistance	
5	Severe disability; bedridden, incontinent, and requires constant nursing care and attention	

5.1) Modified Rankin Score: □

Section VI- Measurements:

5.1	Blood pressure (mmHg)	
5.2	Radial Pulse	□Regular □Irregular
5.3	Height (cm)	
5.4	Waist (cm)	
5.5	Hip (cm)	
5.6	Weight (kg)	
5.7	Finger stick Glucose Reading (mg/dl)	

\

Section V11- Verbal autopsy for suspected stroke related mortalities in the household

Have you had any stroke related mortalities in your household in the last One year?

No □ Yes □

If yes, please answer the following questions.

**S.no**	**Question**	**Code list: Yes…1, No…2**	**Code**
**7.1**	What was you relation to the deceased:		
**7.2**	Did you live with the deceased in the period leading to his/her death?		
**7.3**	When did he/she die?		
**7.4**	Where did the death occur?		
**7.5**	Age of the patient at the time of death:		
**7.6**	What were the events/illness that lead to his/her death?		
**7.7**	What was the cause of death?		
**7.8**	Was he/she ill before death?		
**7.9**	For how long was the patient unwell?		
**7.10**	Did he/she develop weakness on one side of the body prior to death?		
**7.11**	Did the weakness develop suddenly?		
**7.12**	Did it last more than 24 hours?		
**7.13**	Did the deceased have a severe headache prior to death?		
**7.14**	Was it a sudden death (within 24 hours)?		
**7.15**	Was the patient admitted to a hospital/clinic prior to death?		
**7.16**	Was the patient seen by a medical professional?		
**7.17**	For how many nights was he/she admitted?		
**7.18**	Was the deceased hypertensive?		
**7.19**	Was his/her blood pressure under control (<140/90. <130/80 in DM)		
**7.20**	Was the deceased diabetic?		
**7.21**	Did the patient have convulsions prior to death?		
**7.22**	Did he/she have fever/cough/SOB/chest pain prior to death?		
**7.23**	Any other illness?		
**7.24**	Injury or accident?		
**7.25**	Death certificate date of issue and death:		
**7.26**	Cause of death on death certificate:		
**7.27**	Any hospital records prior to death:		
