# Supplementary material for: The burden of stroke and transient ischemic attack in Pakistan: a community-based prevalence study
Source: BMC Neurol. 2009 Dec 1;9:58. doi: 10.1186/1471-2377-9-58 (PMC2793240; doi:10.1186/1471-2377-9-58)
Supplement: Additional file 2 — Supplement 2. The Burden of Stroke and Transient Ischemic Attacks in Pakistan: a Community-based Prevalence Study (Urdu). [file 1471-2377-9-58-S2.PDF]

					رجسٹریشن نمبر:

Prevalence of Stroke and Transient Ischemic Attacks and assessment of risk factors in a community setting in Karachi, Pakistan

سوالنامہ

سیکشن:I

سیریل	درجہ	نام
1.1	جواب دینے والے کا نام	
1.2	گھر کا نمبر	☐☐☐☐ - ☐☐☐☐
1.3	ٹیلفون نمبر	
1.4	جواب کون دے رہا ہے	1 مریض<br>2 رشتہ دار<br>3 کوئی اور وضاحت
1.5	تاریخ پیدائش	☐☐☐☐ سال ☐☐ مہینہ ☐☐ تاریخ
1.6	عمر	
1.7	جنس	1 مرد<br>2 عورت
1.8	قومیت	1 پٹھان<br>2 پنجابی<br>3 سندھی<br>4 بلوچی<br>5 افغانی<br>کوئی اور وضاحت
1.9	کس ہاتھ کو زیادہ استعمال کرتے ہیں؟	1 دائیں<br>2 بائیں
1.10	تعلیم	1 ان پڑھ<br>2 دینی تعلیم<br>3 اسکول کی تعلیم<br>4 سال کی وضاحت

1.11	پیشہ	
1.12	ازدواجی حیثیت	1 شادی شدہ<br>2 غیر شادی شدہ<br>3 طلاق یافتہ<br>4 بیوہ

سیکشن: II (فالج سے متعلق سوالنامہ)

سریل	سوالات	کوڈلسٹ	کوڈز
2.1a	کیا آپ کو جسم کے آدھے حصہ میں کبھی اچانک کمزوری یا فالج کا اثر ہوا ہے؟	1 ہاں<br>2 نہیں<br>99 معلوم نہیں	
2.1b	اگر ہاں تو مختصراً اپنی کیفیت کا حال اور دورانیہ منٹ بیان کیجئے		
2.1c	کیا ان علامات کا کسی ڈاکٹر نے علاج کیا ہے	1 ہاں<br>2 نہیں<br>99 معلوم نہیں	
2.2a	کیا کبھی آپ کے منہ کا کونہ اچانک خود لٹک گیا اور خود واپس نہیں آیا؟ کیا منہ آدھا ٹیڑا ہو گیا تھا؟	1 ہاں<br>2 نہیں<br>99 معلوم نہیں	
2.2b	اگر ہاں تو مختصراً اپنے علامات اور اُنکا دورانیہ منٹ میں بیان کیجئے		
2.2c	کیا ان علامات کا کسی ڈاکٹر نے علاج کیا	1 ہاں<br>2 نہیں<br>99 معلوم نہیں	
2.3a	کیا کبھی اچانک آپ کی آواز میں لُکنت پیدا ہوئی یا آپ بات نہ کر سکتے ہوں؟	1 ہاں<br>2 نہیں<br>99 معلوم نہیں	
2.3b	اگر ہاں تو مختصراً اپنے علامات اور اُنکا دورانیہ بیان کیجئے		
2.3c	کیا ان علامات کا کسی ڈاکٹر نے علاج کیا	1 ہاں<br>2 نہیں<br>99 معلوم نہیں	
2.4a	کیا آپ کے جسم کے آدھے حصے ہاتھ یا پاؤں میں کبھی اچانک سُن پن یا احساس کی کمی (جیسے سو گیا ہو) محسوس ہوا ہے؟	1 ہاں<br>2 نہیں<br>99 معلوم نہیں	

2.4b	اگر ہاں تو مختصراً اپنے علامات اور اُنکا دورانیہ منٹ میں بیان کیجئے		
2.4c	کیا ان علامات کا کسی ڈاکٹر نے علاج کیا	1 ہاں<br>2 نہیں<br>99 معلوم نہیں	
2.5a	کیا آپ کی بینائی میں کبھی اچانک فرق پڑا ہے یہ کمزوری آئی ہے؟ یہ آپ کو دو نظر آیا ہے؟ یہ اندھیرا آیا ہے؟ تصوریں دیکھ کر بتائیں؟	1 ہاں<br>2 نہیں<br>99 معلوم نہیں	
2.5b	اگر ہاں تو مختصراً اپنے علامات اور اُنکا دورانیہ منٹ میں بیان کیجئے		
2.5c	کیا ان علامات کا کسی ڈاکٹر نے علاج کیا	1 ہاں<br>2 نہیں<br>99 معلوم نہیں	
2.6a	کیا کبھی کسی ڈاکٹر نے آپ کو یہ کہا ہے کہ آپ کو فالج کا دورہ ہوا ہے؟	1 ہاں<br>2 نہیں<br>99 معلوم نہیں	
2.6b	اگر ہاں تو مختصراً اپنے علامات اور اُنکا دورانیہ منٹ میں بیان کریں		
2.6c	کیا ان علامات کا کسی ڈاکٹر نے علاج کیا	1 ہاں<br>2 نہیں<br>99 معلوم نہیں	

سیکشن: III (علاماتی سوالنامہ)

سیریل	سوالات	کوڈ لسٹ	کوڈز
3.a.1	کیا پچھلے 12 مہینوں میں آپ کے جسم کے کسی حصہ میں اچانک سُن پن یا سنناہٹ محسوس ہوئی اگر نہ ہے تو اگلے سوالنامے پر جائیں۔ اگر ہاں ہے تو مندرجہ ذیل سوالات کے جواب دیجئے۔	1 ہاں<br>2 نہیں<br>99 معلوم نہیں	
3.a.2	مختصر اپنی کیفیت بیان کیجئے۔		

3.a.3	آپ کے کس ہاتھ پاؤں یا چہرے پر اثر ہوا؟	1 دائیں طرف کا چہرہ<br>2 دائیں طرف کا ہاتھ<br>3 دائیں طرف کا بازو<br>4 دائیں طرف کا ٹانگ<br>5 دائیں طرف کا پاؤں<br>6 بائیں طرف کا چہرہ<br>7 بائیں طرف کا ہاتھ<br>8 بائیں طرف کا بازو<br>9 بائیں طرف کا ٹانگ<br>10 بائیں طرف کا پاؤں	ہاں نہیں<br>☐ ☐<br>☐ ☐<br>☐ ☐<br>☐ ☐<br>☐ ☐<br>☐ ☐<br>☐ ☐<br>☐ ☐<br>☐ ☐<br>☐ ☐
3.a.4	کیا آپ کے بازو یا ٹانگ میں سُن ہونے کے ساتھ درد بھی ہوا	1 ہاں<br>2 نہیں<br>99 معلوم نہیں	
3.a.5	اس قسم کے آپ کو کتنے دورے پڑے	1 ایک دفعہ<br>2 دو دفعہ<br>3 تین سے پانچ دفعہ<br>4 پانچ سے زیادہ دفعہ	
3.a.6	پہلا کب ہوا	☐☐ ☐☐ ☐☐☐☐	
3.a.7	اگر ایک سے زیادہ تو آخری کب پڑا	☐☐ ☐☐ ☐☐☐☐	
3.a.8	اگر ایک سے زیادہ تو کب دورے پڑے	1 دن میں کئی مرتبہ<br>2 دن میں صرف ایک مرتبہ<br>3 ہفتے میں کئی مرتبہ<br>4 ہفتے میں ایک مرتبہ<br>5 مہینہ میں کئی مرتبہ<br>6 سال میں ایک کمرتبہ	
3.a.9	دورہ اندازً کتنے وقفہ کیلیئے ہوا	1 پانچ منٹ سے کم<br>2 پانچ منٹ سے ایک گھنٹے تک<br>3 ایک سے چھ گھنٹے تک<br>4 چھ سے چوبیس گھنٹے تک<br>5 ایک دن سے زیادہ	

3.a.10	کیا درج ذیل میں بھی کوئی اثرات آپ کو سُن ہونے کے ساتھ محسوس ہوئے۔	1 فالج<br>2 بولنے میں مشکل<br>3 بیہوش ہونا<br>4 سخت سر درد<br>5 بینائی کا کھو جانا<br>6 مرگی جیسے دورے پڑنا<br>7 چکر آنا<br>8 گھبراہٹ<br>9 سُن ہونا	ہاں ☐ نہیں ☐<br>☐ ☐<br>☐ ☐<br>☐ ☐<br>☐ ☐<br>☐ ☐<br>☐ ☐<br>☐ ☐<br>☐ ☐<br>☐ ☐
3.a.11	کیا آپ نے سُن پن کیلئے کسی ڈاکٹر کو دیکھایا ہے۔	1 ہاں<br>2 نہیں	

سیکشن: III B (کمزوری)

سیریل	سوالات	کوڈلسٹ	کوڈز
3.B.1	کیا پچھلے 12 مہینوں میں آپ کے ٹانگ، پیر، بازو یا ہاتھ میں اچانک کمزوری / فالج محسوس ہوا؟	1 اگر ہاں تو مندرجہ ذیل سوالات کے جواب دیجئے۔<br>2 اگر نہیں یا پتہ نہیں ہے تو اگلے سوالنامہ پر جائیں	
3.B.2	مختصراً اپنی کیفیت بیان کریجئے		
3.B.3	کس ہاتھ پاؤں بازو یا ٹانگ پا اثر ہوا؟	1 دائیں طرف کا چہرہ<br>2 دائیں طرف کا ہاتھ<br>3 دائیں طرف کا بازو<br>4 دائیں طرف کا ٹانگ<br>5 دائیں طرف کا پاؤں<br>6 بائیں طرف کا چہرہ<br>7 بائیں طرف کا ہاتھ<br>8 بائیں طرف کا بازو<br>9 بائیں طرف کا ٹانگ<br>10 بائیں طرف کا پاؤں	ہاں نہیں<br>☐ ☐<br>☐ ☐<br>☐ ☐<br>☐ ☐<br>☐ ☐<br>☐ ☐<br>☐ ☐<br>☐ ☐<br>☐ ☐<br>☐ ☐
3.B.4	کیا کمزوری کے ساتھ آپ کو درد بھی محسوس ہوا؟	1 ہاں<br>2 نہیں<br>99 معلوم نہیں	

3.B.5	اس قسم کے آپ کو کتنے دورے محسوس ہوئے؟	1	ایک دفعہ		
		2	دو دفعہ		
		3	تین سے پانچ دفعہ		
		4	پانچ سے زیادہ دفعہ		
3.B.6	پہلا کب ہوا		☐☐ ☐☐ ☐☐☐☐		
3.B.7	اگر ایک سے زیادہ تو آخری کب ہوا		☐☐ ☐☐ ☐☐☐☐		
3.B.8	اگر ایک سے زیادہ تو کب دورے پڑے	1	دن میں صرف ایک مرتبہ		
		2	دن میں کئی مرتبہ		
		3	ہفتے میں ایک مرتبہ		
		4	ہفتے میں کئی مرتبہ		
		5	مہینہ میں کئی مرتبہ		
		6	سال میں کئی مرتبہ		
3.B.9	دورہ اندازاً کتنے وقفہ کیلئے ہوا	1	پانچ منٹ سے کم		
		2	پانچ منٹ سے ایک گھنٹے تک		
		3	ایک سے چھ گھنٹے تک		
		4	چھ سے چوبیس گھنٹے تک		
		5	ایک دن سے زیادہ		
3.B.10	کیا کمزوری اور فالج کے علاوہ آپ کو مندرجہ زیل علامات میں سے بھی کوئی محسوس ہوئے			ہاں	نہیں
		1	فالج	☐	☐
		2	بولنے میں مشکل	☐	☐
		3	بیہوش ہونا	☐	☐
		4	سخت سردرد	☐	☐
		5	بینائی کا کھو جانا	☐	☐
		6	مرگی جیسے دورے پڑنا	☐	☐
		7	چکر آنا	☐	☐
		8	گھبراہٹ	☐	☐
		9	سُن ہونا	☐	☐
3.B.12	کیا آپ نے اس کمزوری/ فالج کے لئے کسی ڈاکٹر ہو دیکھایا	1	ہاں		
		2	نہیں		
		99	معلوم نہیں		

سیکشن: C III (بینائی)

سیریل	سوالات	کوڈلسٹ	کوڈز
3.C.1	کیا پچھلے 12 مہینوں میں آپ کو اچانک تھوڑی دیر کے لئے اپنی بینائی کے جانے کا یا دُھندلا نظر آنے کا احساس ہوا؟	1 اگر ہاں ہے تو مندرجہ زیل سوالات کے جواب دیجئے<br>2 اگر نہیں ہے تو اگلے سوال نامے پر جائیں<br>3 معلوم نہیں	
3.C.2	مختصراً اپنی کیفیت بیان کریجئے۔		
3.C.3	آپ کی بینائی کا کونسہ حصہ اثر پزیر ہوا؟	1 دائیں آنکھ<br>2 بائیں آنکھ<br>3 دونوں آنکھیں<br>4 دائیں طرف کی بینائی<br>5 بائیں طرف کی بینائی	
3.C.4	آپ کے کتنی مرتبہ بینائی کے جاتے رہنے یا دُھندلا نظر آنے کے دورے پڑے	1 ایک دفعہ<br>2 دو دفعہ<br>3 تین سے پانچ دفعہ<br>4 پانچ سے زیادہ دفعہ	
3.C.5	پہلا کب ہوا	☐☐ ☐☐ ☐☐☐☐	
3.C.6	اگر ایک سے زیادہ تو آخری کب ہوا	☐☐ ☐☐ ☐☐☐☐	
3.C.7	اگر ایک سے زیادہ تو کب کب دورے پڑے	1 دن میں کئی مرتبہ<br>2 دن میں صرف ایک مرتبہ<br>3 ہفتے میں کئی مرتبہ<br>4 ہفتے میں ایک مرتبہ<br>5 مہینہ میں کئی مرتبہ<br>6 سال میں ایک کمرتبہ	
3.C.8	ان دوروں کی مُدت کیا تھی	1 پانچ منٹ سے کم<br>2 پانچ منٹ سے ایک گھنٹے تک<br>3 ایک سے چھ گھنٹے تک<br>4 چھ سے چوبیس گھنٹے تک<br>5 ایک دن سے زیادہ	

3.C.9	کیا درج ذیل میں بھی کوئی اثرات آپ کو بینائی کے خلل کے دوران محسوس ہوئے۔	1 فالج<br>2 بولنے میں مشکل<br>3 بے ہوش ہونا<br>4 سخت سر درد<br>5 بینائی کا کھو جانا<br>6 مرگی جیسے دورے پڑنا<br>7 چکر آنا<br>8 گھبراہٹ<br>9 سُن ہونا	ہاں ☐ نہیں ☐<br>☐ ☐<br>☐ ☐<br>☐ ☐<br>☐ ☐<br>☐ ☐<br>☐ ☐<br>☐ ☐<br>☐ ☐
3.C.10	کیا آپ نے بینائی کے لئے کسی ڈاکٹر کو دیکھایا؟	1 ہاں<br>2 نہیں<br>99 معلوم نہیں	

سیکشن: III D (گویائی)

سیریل	سوالات	کوڈلسٹ	کوڈز
3.D.1	کیا پچھلے ۱۲ مہینوں میں آپ کو بولنے میں کوئی دقت محسوس ہوئی ہے جسکا دورانیہ ۲ منٹ سے زیادہ ہو؟	1 اگر نہیں ہے تو اگلے سوالنامے پر جائیں۔<br>2 اگر ہاں ہے تو مندرجہ ذیل سوالات کے جواب دیجئے<br>3 معلوم نہیں	
3.D.2	مختصراً اپنی کیفیت بیان کریجئے۔		
3.D.3	اگر ہاں ہے تو آپ کو گویائی جانے کے کتنے دورے پڑے	1 ایک دفعہ<br>2 دو دفعہ<br>3 تین سے پانچ دفعہ<br>4 پانچ سے زیادہ دفعہ	
3.D.5	پہلا کب ہوا	☐☐ ☐☐ ☐☐☐☐	
3.D.6	اگر ایک سے زیادہ تو پہلا کب پڑا۔	☐☐ ☐☐ ☐☐☐☐	
3.D.7	اگر ایک سے زیادہ تو کب کب دورے پڑے	1 دن میں کئی مرتبہ<br>2 دن میں صرف ایک مرتبہ<br>3 ہفتے میں کئی مرتبہ<br>4 ہفتے میں ایک مرتبہ<br>5 مہینہ میں کئی مرتبہ<br>6 سال میں کئی مرتبہ	

3.D.8	ان دوروں کا دورانیہ کیا تھا	1 پانچ منٹ سے کم<br>2 پانچ منٹ سے ایک گھنٹے تک<br>3 ایک سے چھ گھنٹے تک<br>4 چھ سے چوبیس گھنٹے تک<br>5 ایک دن سے زیادہ	
3.D.9	کیا درج زیل میں بھی کوئی اثرات آپ کو بینائی کے خلل کے دوران محسوس ہوئے۔	1 فالج<br>2 بولنے میں مشکل<br>3 بیہوش ہونا<br>4 سخت سر درد<br>5 بینائی کا کھو جانا<br>6 مرگی جیسے دورے پڑنا<br>7 چکر آنا<br>8 گھبراہٹ<br>9 سُن ہونا	ہاں نہیں<br>☐ ☐<br>☐ ☐<br>☐ ☐<br>☐ ☐<br>☐ ☐<br>☐ ☐<br>☐ ☐<br>☐ ☐<br>☐ ☐
3.D.10	کیا آپ نے بولنے کی دقت کے لئے کسی ڈاکٹر کو دیکھایا؟	1 ہاں<br>2 نہیں<br>99 معلوم نہیں	

سیکشن: III E (چکر آنا)

سیریل	سوالات	کوڈلسٹ	کوڈز
3.E.1	کیا پچھلے ۱۲مہینوں میں آپکو اچانک چکر آئے یا چلنے میں دُشواری ہوئی یہ سر کا ہلکا پن یا توازن رکھنے میں کوئی دقت محسوس ہوئی ؟	1 اگر ہاں ہے تو مندرجہ زیل سوالات کے جواب دیجئے<br>2 اگر نہیں ہے تو اگلے سوالنامے پر جائیں<br>3 معلوم نہیں	
3.E.2	مختصراً اپنی کیفیت بیان کریجئے۔		
3.E.3	اگر ہاں ہے تو مندرجہ زیل سوالات کے جوابات دیں	1 چکر آنا<br>2 توازن رکھنے مین دُشواری<br>3 گھوم جانے کا احساس۔<br>4 چلنے پھرنے میں دُشواری<br>5 بے ہوش ہوجانا	ہاں نہیں<br>☐ ☐<br>☐ ☐<br>☐ ☐<br>☐ ☐<br>☐ ☐
3.E.4	پچھلے ۱۲مہینوں میں کتنی مرتبہ دورے پڑے ہیں	1 ایک دفعہ<br>2 دو دفعہ<br>3 تین سے پانچ دفعہ<br>4 پانچ سے زیادہ دفعہ	

3.E.5	پہلا کب ہوا	☐☐ ☐☐ ☐☐☐☐	
3.E.6	اگر ایک سے زیادہ تو آخری دورہ کب پڑا	☐☐ ☐☐ ☐☐☐☐	
3.E.7	اگر ایک سے زیادہ تو کب کب دورے پڑے	1 دن میں کئی مرتبہ<br>2 دن میں صرف ایک مرتبہ<br>3 ہفتے میں کئی مرتبہ<br>4 ہفتے میں ایک مرتبہ<br>5 مہینہ میں کئی مرتبہ<br>6 سال میں ایک کم مرتبہ	
3.E.8	ان دوروں کی مُدت کیا تھی	1 پانچ منٹ سے کم<br>2 پانچ منٹ سے ایک گھنٹے تک<br>3 ایک سے چھ گھنٹے تک<br>4 چھ سے چوبیس گھنٹے تک<br>5 ایک دن سے زیادہ	
3.E.9	کیا درج زیل میں بھی کوئی اثرات آپ کو بینائی کے خلل کے دوران محسوس ہوئے۔		ہاں / نہیں
		1 فالج	☐ ☐
		2 بولنے میں مشکل	☐ ☐
		3 بیہوش ہونا	☐ ☐
		4 سخت سر درد	☐ ☐
		5 بینائی کا کھو جانا	☐ ☐
		6 مرگی جیسے دورے پڑنا	☐ ☐
		7 چکر آنا	☐ ☐
		8 گھبراہٹ	☐ ☐
		9 سُن ہونا	☐ ☐
3.E.10	کیا آپ نے کسی ڈاکٹر ہو دیکھایا	1 ہاں<br>2 نہیں<br>99 معلوم نہیں	

سیکشن IV: (مکانی محرکات کا جائزہ)

سیریل	سوالات	کوڈلسٹ	کوڈ
4.1	کیا آپ کو کبھی فالج ہوا ہے؟	1 ہاں<br>2 نہیں<br>99 معلوم نہیں	
4.2	کیا آپ کو کبھی فالج ہوا ہے جو ۲۴ گھنٹوں میں ختم ہو گیا ہو؟	1 ہاں<br>2 نہیں<br>99 معلوم نہیں	
4.3	کیا کبھی آپ کے دل کی دھڑکن غیر معتدل ہوئی؟	1 ہاں<br>2 نہیں<br>99 معلوم نہیں	
4.4	کیا آپکو شگر کی بیماری ہے؟	1 ہاں<br>2 نہیں<br>99 معلوم نہیں	
4.5	کیا آپ اسکے لئے دوا استعمال کرتے ہیں؟	1 ہاں<br>2 نہیں<br>99 معلوم نہیں	
4.6	کیا آپکو بلڈ پریشر ہے؟	1 ہاں<br>2 نہیں<br>99 معلوم نہیں	
4.7	کیا آپ نے کبھی اپنا بلڈ پریشر چیک کرایا ہے؟	1 ہاں<br>2 نہیں<br>99 معلوم نہیں	
4.8	اگر ہاں تو کتنا تھا؟	□□□ / □□□	
4.9	کیا آپ بلڈ پریشر کیلیئے کوئی دوا لیتے ہیں؟	1 ہاں<br>2 نہیں<br>99 معلوم نہیں	
4.10	کیا آپ تمباکو نوشی کرتے ہیں؟	1 ہاں<br>2 نہیں<br>99 معلوم نہیں	
4.11	اگر ہاں تو کتنی	□□□	

4.12	اگر چھوڑی تو کتنے عرصہ پہلے؟	☐☐	
4.13	کیا آپ کو کولسٹرول زیادہ رہتا ہے؟	1 ہاں<br>2 نہیں<br>99 معلوم نہیں	
4.14	کبھی آپکو جھٹکے آئے ہیں؟	1 ہاں<br>2 نہیں<br>99 معلوم نہیں	
4.15	کبھی آپ کو گھبراہٹ یا سینے میں بوجھ ہوا ہے؟	1 ہاں<br>2 نہیں<br>99 معلوم نہیں	
4.16	اگر ہاں تو آرام کے دوران یا مشقت پر؟	1 ہاں<br>2 نہیں<br>99 معلوم نہیں	
4.17	زیادہ چلنے یا کام کرنے سے سینے میں درد ہوا تھا	1 ہاں<br>2 نہیں<br>99 معلوم نہیں	
4.18	کیا آپکو کبھی دل کا دورہ پڑا ہے؟	1 ہاں<br>2 نہیں<br>99 معلوم نہیں	
4.19	کیا آپ کا کبھی دل کا آپریشن ہوا ہے۔	1 ہاں<br>2 نہیں<br>99 معلوم نہیں	
4.20	کیا آپکو کبھی جوڑوں کے درد کے ساتھ بخار آیا؟	1 ہاں<br>2 نہیں<br>99 معلوم نہیں	
4.21	کیا آپکو دل کے Valve کی بیماری ہے؟	1 ہاں<br>2 نہیں<br>99 معلوم نہیں	
4.22	کیا آپ کے دل کا Valve بدلا گیا؟	1 ہاں<br>2 نہیں<br>99 معلوم نہیں	

	1 ہاں<br>2 نہیں<br>99 معلوم نہیں	کیا چلنے سے آپ کے ٹانگوں میں درد ہوتا ہے؟	4.23
	1 ہاں<br>2 نہیں<br>99 معلوم نہیں	کیا رُکتے ہی درد غائب ہو جاتا ہے؟	4.24
	1 ہاں<br>2 نہیں<br>99 معلوم نہیں	کیا آپ کو ہر دفعہ چلنے پر یہ درد ہوتا ہے؟	4.25
	1 ہاں<br>2 نہیں<br>99 معلوم نہیں	کیا آپ شراب پیتے ہیں	4.26
		عام طور پر کتنی شراب پیتے ہیں	4.27
		آپ نے آخری بار شراب کب چکھی تھی	4.28
	1 ہاں<br>2 نہیں<br>99 معلوم نہیں	کیا آپ پان، سُپاری یا گُٹکا استعمال کرتے ہیں؟	4.29
	1 ہاں<br>2 نہیں<br>99 معلوم نہیں	کیا آپ کی ماہواری بند ہو چُکی ہے؟	4.30
	**Title of the scale**	**Berlin Scale for apnea**	
		**درجہ اول (Category 1)**	
	1 ہاں<br>2 نہیں<br>99 معلوم نہیں	کیا آپ خراٹے لیتے ہیں؟	4.31
	1 سانس لینے سے تھوڑی زیادہ<br>2 باتوں کی طرح بلند<br>3 باتوں سے زیادہ بلند<br>4 اتنا بلند کہ ساتھ والے کمرے میں سنائی دے	اگر آپ خراٹے لیتے ہیں تو	4.32

4.33	کتنی دفعہ	1 روزانہ<br>2 ہفتے تین سے چار مرتبہ<br>3 ہفتے میں ایک سے دو مرتبہ<br>4 مہینے میں ایک سے دو مرتبہ<br>5 کبھی کبھار	
4.34	کیا کبھی دوسرے لوگ متاثر ہوئے ہیں؟	1 ہاں<br>2 نہیں<br>99 معلوم نہیں	
4.35	کیا خراٹے لینے کے دوران آپ کی سانس روک جاتی ہے؟	1 روزانہ<br>2 ہفتے میں تین سے چار مرتبہ<br>3 ہفتے میں ایک سے دو مرتبہ<br>4 مہینے میں ایک سے دو مرتبہ<br>5 کبھی کبھار	
4.36	درجہ دوم (Category 2)		
4.37	کیا آپ نیند کے بعد تھکاوٹ محسوس کرتے ہیں؟	1 روزانہ<br>2 ہفتے میں تین سے چار مرتبہ<br>3 ہفتے میں ایک سے دو مرتبہ<br>4 مہینے میں ایک سے دو مرتبہ<br>5 کبھی کبھار	
4.38	کیا آپ جاگتے ہوئے تھکاوٹ محسوس کرتے ہیں؟	1 روزانہ<br>2 ہفتے میں تین سے چار مرتبہ<br>3 ہفتے میں ایک سے دو مرتبہ<br>4 مہینے میں ایک سے دو مرتبہ<br>5 کبھی کبھار	
4.39	کیا آپ کبھی گاڑی چلاتے ہوئے سو گئے یہ غشی محسوس ہوئی؟	1 ہاں<br>2 نہیں<br>99 معلوم نہیں	
4.40	ایسا کتنی بار ہوا؟	1 روزانہ<br>2 ہفتے میں تین سے چار مرتبہ<br>3 ہفتے میں ایک سے دو مرتبہ<br>4 مہینے میں ایک سے دو مرتبہ<br>5 کبھی کبھار	

4.41	درجہ سوم (Catgory 3)			
4.42	کیا آپ کا بلڈ پریشر زیادہ رہتا ہے؟	1 ہاں<br>2 نہیں<br>99 معلوم نہیں		
	اگر آپ کے خاندان میں کسی کو بھی مندرجہ زیل بیماری ہوئی ہو تو اُسکی نشان دہی کیجئے؟	1. اول درجے رشتے داروں کی تعداد	2. کم ترین عمر	
4.43	فالج			
4.44	عارضی فالج			
4.45	شگر کی بیماری			
4.46	ہائی بلڈ پریشر			
4.47	جھٹکے آنا			
4.48	دل کے درد کی تکلیف			
4.49	قبل از وقت دل کی بیماری (مرد:55 سال یا کم،عورت 65 سال یا کم)			

سیکشن:V (معزوری کا تجزیہ)

سیریل	وضاحت	
0	کوئی علامات نہیں	
1	باوجود علامات کے کوئی خاص معزوری نہیں، روزمرع کے کام کرنے میں کوی مشکل نہیں	
2	تھوری معزوری، پچھلے تمام کام کرنے میں دشواری،لیکن اپنی دیکھ بھال کرنے کے قابل	
3	درمیانی معزوری، معمولی سی مدد کی ضرورت لیکن اس قابل کہ مدد کے بغیر چل سکے	
4	درمیانی شدید معزوری، بغیر مدد کے چلنے پھرنے سے قاصر اپنی جسمانی ضروریات بھی خود پوری کرنے سے قاصر ہو۔	
5	شدید معزوری، بستر سے مستقل لگا ہوا، مستقل دیکھ بھال اور نگہداشت کا محتاج	

سیکشن: VI اسکور

5.1	بلڈ پریشر (mmHg)	/
5.2	نبض روزانہ کبھی کبھی	
5.3	قد	
5.4	کمر	
5.5	کولھے	

5.6	وزن		
5.7	اُنگلی سے شکر کی مقدار		
5.8	اُنگلی سے کولیسٹرول کی مقدار		

سیکشن: VII آپ کے گھر میں کوئی ایسا فرد ہے جو فالج کے باعث معزور ہو اور یہاں نہیں آ سکا؟

کیا پچھلے ایک سال میں آپ کے گھر میں فالج سے متعلق کوئی موت واقع ہوئی ہے؟ ہاں ☐ نہیں ☐

اگر ہاں تو براہ مہربانی مندرجہ زیل سوالات کا جواب دیجئے

سیریل	سوالات	کوڈلسٹ	کوڈز
7.1	مرحوم سے آپکا رشتہ	1 ماں<br>2 والد<br>3 بھائی<br>4 بہن<br>5 شوہر<br>6 بیوی<br>7 کوئی اور وضاحت	
7.2	کیا آپ انکے ساتھ رہتے تھے		
7.3	انکا انتقال کب ہوا	/ /	
7.4	کہاں ہوا		
7.5	اُن کی عمر کیا تھی	سال	
7.6	اُن کی موت کے کیا اسباب تھے		
7.7	موت کی وجہ کیا تھی		
7.8	کیا وہ موت سے پہلے بیمار تھے		
7.9	کتنے عرصہ بیمار تھے		
7.10	کیا اُن کے جسم کے آدھے حصے میں کمزوری ہوئی تھی		
7.11	کیا یہ کمزوری اچانک ظاہر ہوئی		
7.12	کیا یہ کمزوری 24 گھنٹے سے زیادہ رہی		
7.13	کیا سر میں شدید درد ہوا تھا		
7.14	کیا موت اچانک واقع ہوئی		
7.15	کیا وہ کسی ہسپتال میں داخل تھے		

			کیا کسی ڈاکٹر نے دیکھا تھا	7.16
			ہسپتال میں کتنے دن داخل رہے	7.17
			کیا اُن کا بلڈ پریشر بڑا ہوا تھا	7.18
			کیا بلڈ پریشر قابو میں تھا	7.19
			کیا مرحوم کو شگر کی بیماری تھی	7.20
			کیا مریض کو دورے پڑتے تھے	7.21
			کیا بخار، کھانسی یا سانس کی تکلیف تھی	7.22
			کوئی اور بیماری تھی	7.23
			کوئی چوٹ یا ایکسیڈینٹ	7.24
			موت واقع ہونے کی تاریخ اور سرٹیفکیٹ	7.25
			سرٹیفکیٹ میں موت کی وجہ	7.26
			موت سے قبل ہسپتال کا کوئی ریکارڈ	7.27
