# Supplementary figures and images for: The burden of stroke and transient ischemic attack in Pakistan: a community-based prevalence study
Source: BMC Neurol. 2009 Dec 1;9:58. doi: 10.1186/1471-2377-9-58 (PMC2793240; doi:10.1186/1471-2377-9-58)

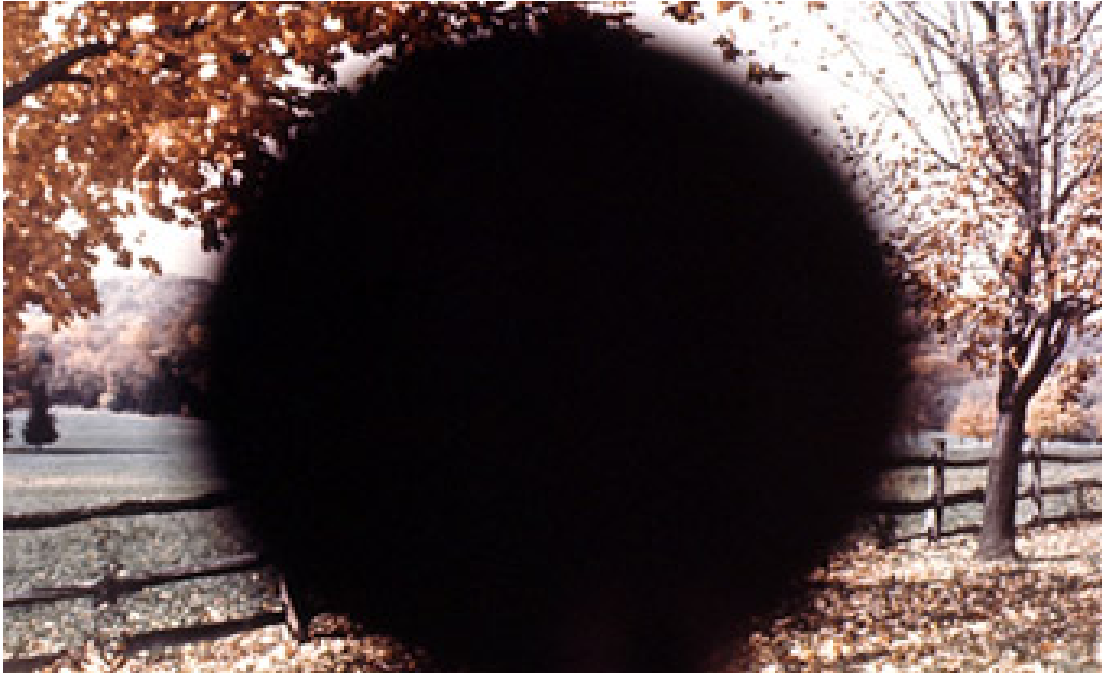

Central Scotoma

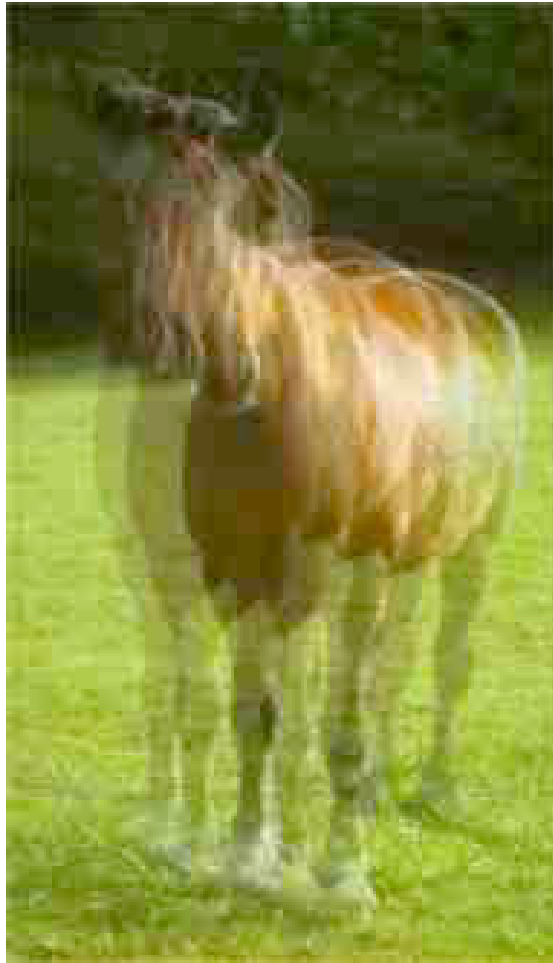

**Triplopia**

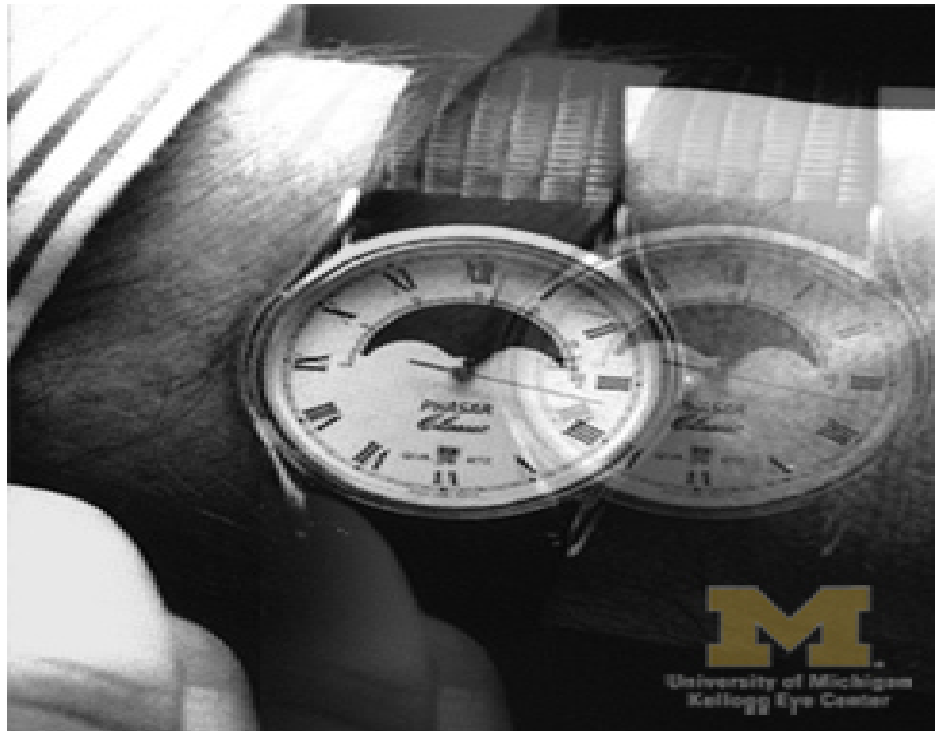

**Diplopia**

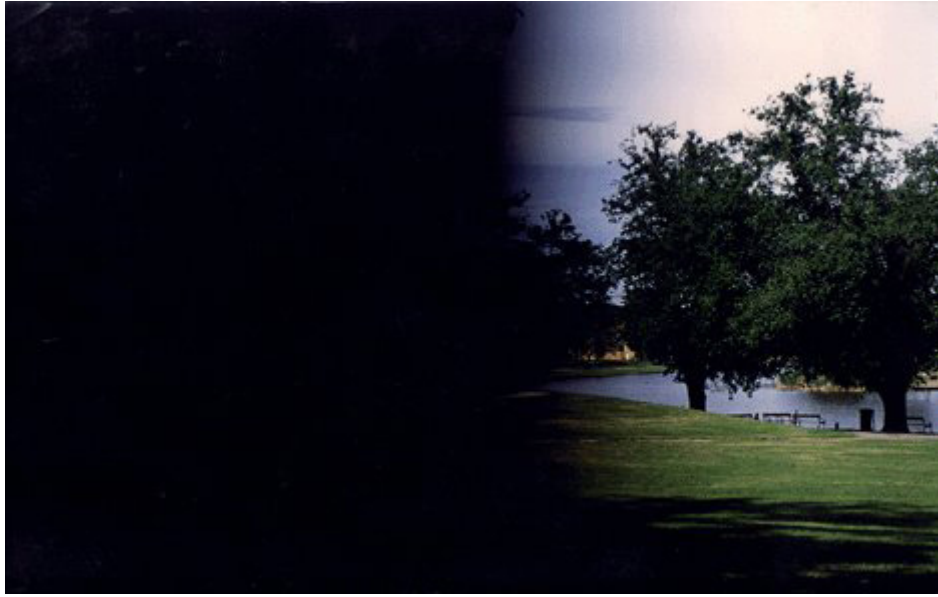

**Hemifield Loss**

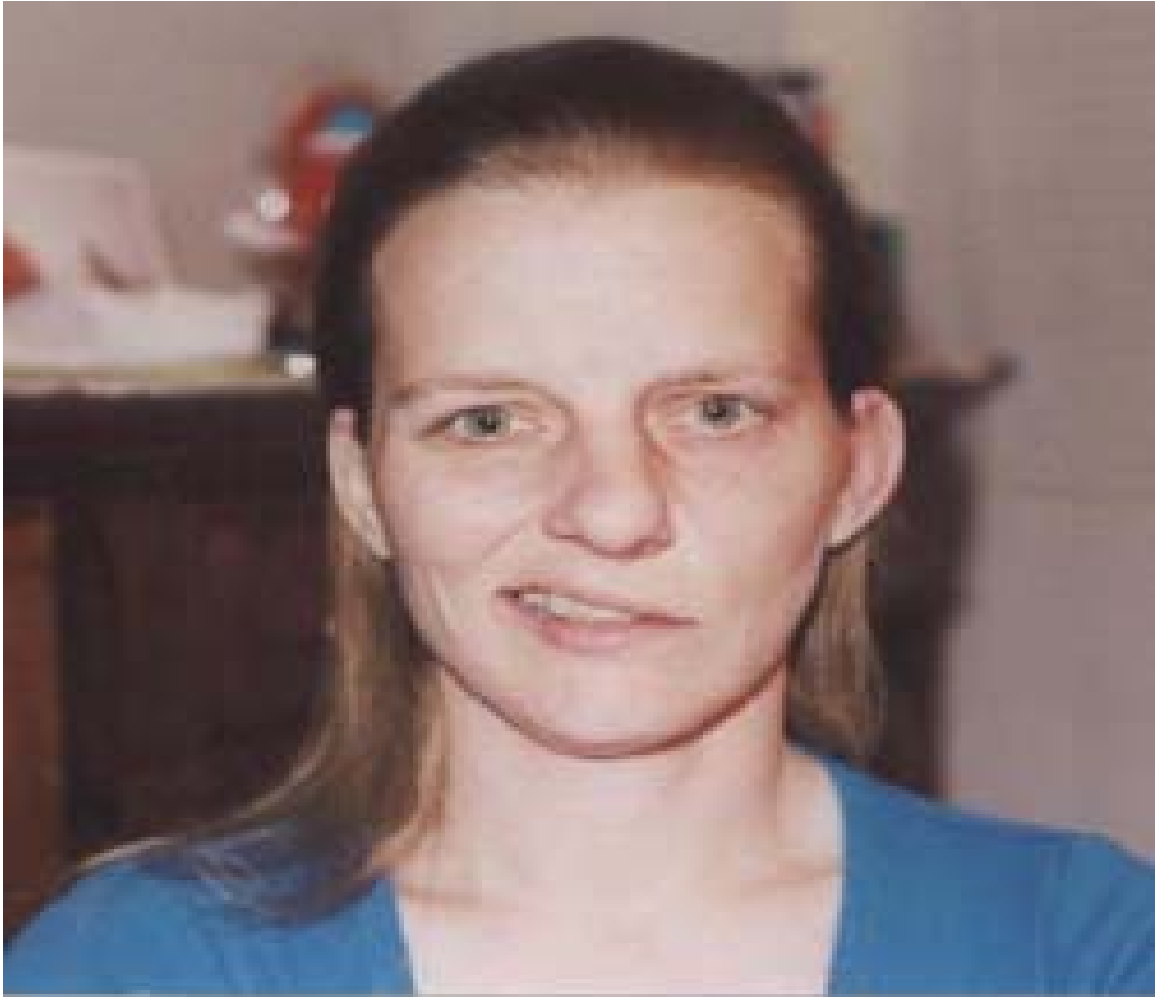

**Facial Paresis**

Supplement: Additional file 3 — Supplement 3. This PDF contains the visual aids used in the study. [file 1471-2377-9-58-S3.PDF]
